# 

**Published:** 1876-01

**Authors:** 


### CONTENTS.

PAGE.

**CONTRIBUTIONS.**

Gastronomics. Ben. H. Pratt....85
Our National Disease. P. H. Hayes, M. D....87
A Human Monster. A. R. Kilpatrick, M. D....88
Dr. Ned's Reflections. Dr. Ned....90

**MEDICAL ITEMS AND NEWS.**

Hysteria—Dysentery—Pruritis of Variola—Cerebral Rheumatism—Scirrhus of Breast—Eczema Rubrum—Intermittent Fever—Anti-Catarrhal Pills—Passage of Pins per Anum—Belgian Medical Laws—Cardiac Œdema—Nickelization—Pityriasis Capatis—Sore Throat—Pruritis—Traumatic Erysipelas—Diphtheria—Fish Diet—Biliary Calculi—Pneumonia—Parisian Police Lanterns—Mucus Polypus—Burns—Wet Strapping—Too Much Tobacco—Inunction—Chorea—Cod Liver Oil Formula—Insufflation in Intestinal Obstruction—WinterCough—Cystic Paralysis—Boils—Tippling—Common Cold—Typhoid Fever—Respiratory Percussion—Diarrhœa of Phthisis....93 to 100

**OUR CORRESPONDENCE.**

Four Letters with Replies....100

PAGE.

**HOUSEHOLD HINTS AND RECIPES.**

The Preserve Posts—Pocket Mucilage—Cement for Iron—Marking Tools—Electroplating—To Dye Ivory Blue—Improved Starch—To Preserve a Bouquet—Milk Kept by Chloroform—Ammonia Fumes on Flowers—To Extinguish Lamps—Coffee Making—Delicacies for the Sick—Chapped Hands—Harness Polish—Hair Restorer—Stencil Ink—Washing Blue—Adhesive Paper—Handles to Prevent Splitting—Vegetable Marrow Preserve—Bag Marking Ink—Mildew, to Remove—Waterproofing Woven Fabrics—Milk of Roses—Copper-Coating Castings—Honey, to Improve—Flowers, to Freshen....101 to 105

**EDITORIAL.**

Doctor Making....105
Glaucoma, Artificial Pupil....105
Stomachs....107
Pleasure and Iron....108

**EDITORIAL CHIT-CHAT.**

Fifty-five Cents—James Vick—Russian Overcoat—Encyclopedias—That Story—Dr. W. C. Doane—The Reminder—A Strong Letter—Park Church Fair—Quack Almanacs—Copenhagen—The Surgical Institute—Feelingly—Compliments of the Season—Christmas—Hooked Adder in Stomach..109 to 113

